# Trends and regional variations in chronic ischemic heart disease and lung cancer-related mortality among American adults: Insights from retrospective CDC wonder analysis

**DOI:** 10.1016/j.ijcrp.2025.200377

**Published:** 2025-02-14

**Authors:** Eman Ali, Hafsah Alim Ur Rahman, Usama Hussain Kamal, Muhammad Ahmed Ali Fahim, Madiha Salman, Afia Salman, Hamza Nawaz Khan, Farah Yasmin, Chmsalddin Alkhas, Afsana Ansari Shaik, Muhammad Sohaib Asghar, M. Chadi Alraies

**Affiliations:** a Institute: Dow University of Health Sciences, Karachi, Pakistan; b Institute: Services Institute of Medical Sciences, Lahore, Pakistan; c Institute: Dow Medical College, Dow University of Health Sciences, Karachi Pakistan; d Institute: Yale School of Medicine, New Haven, CT, USA; e Institute: Cardiovascular Research Department, Harper University Hospital, Detroit, MI, USA; f Institute: Division of Nephrology and Hypertension, Mayo Clinic Rochester, MN, USA; g Institute: Department of Internal Medicine, AdventHealth Sebring, FL, USA; h Institute: Cardiovascular Institute, Detroit Medical Center, DMC Heart Hospital, 311 Mack Ave, Detroit, MI, 48201, USA

**Keywords:** CDC, Mortality, Ischemic heart disease

## Abstract

**Introduction:**

Lung cancer remains the leading cause of cancer-related mortality in the United States and shares cardiovascular risk factors with chronic ischemic heart disease (CIHD). However, the cumulative mortality burden of these comorbid conditions is underexplored. This study aims to retrospectively assess mortality trends among American adults with concurrent lung cancer and CIHD.

**Methods:**

We utilized death certificate data from the Centers for Disease Control and Prevention's Wide-Ranging Online Data for Epidemiologic Research (CDC WONDER) database, encompassing ICD-10 codes for individuals aged ≥45 years from 1999 to 2020. Age-adjusted mortality rates (AAMRs) per 100,000 population, annual percentage change (APC), and corresponding 95 % confidence intervals (CIs) were calculated. Data were further stratified by year, sex, race, and geographic region (state, rural-urban, and census regions).

**Results:**

A total of 214,785 deaths were identified in adults aged ≥45 years with comorbid lung cancer and CIHD. The overall AAMR between 1999 and 2020 was 8.4 per 100,000 (95 % CI: 8.3 to 8.4). AAMRs remained relatively stable from 1999 to 2005 (APC: −0.84 %; 95 % CI: −1.91 to 1.54), followed by a significant decline from 2005 to 2010 (APC: −2.37 %; 95 % CI: −5.58 to −0.61) and from 2010 to 2017 (APC: −4.72 %; 95 % CI: −7.61 to −3.60). A subsequent period of stability was noted between 2017 and 2020 (APC: 0.86 %; 95 % CI: −2.17 to 5.22). In 1999, men had a threefold higher mortality rate compared to women (AAMR: 17.8 vs. 5.7), with a non-significant decline by 2020 (AAMR: 10 vs. 4). Stratification by race/ethnicity revealed that non-Hispanic (NH) Whites exhibited the highest AAMR at 9.3, followed by NH American Indian or Alaska Natives (7.3), NH Blacks (6.8), Hispanic/Latinos (3.3), and NH Asians or Pacific Islanders (3.2). Geographically, AAMRs were highest in the Midwest (9.6), followed by the Northeast (8.8), South (8.4), and West (6.8). Non-metropolitan regions exhibited higher AAMRs compared to metropolitan areas (10.3 vs. 8.0). States in the top 90th percentile, such as West Virginia, Kentucky, Vermont, Ohio, and Rhode Island, had nearly triple the AAMRs compared to states in the lower 10th percentile, including Utah, Nevada, Arizona, New Mexico, and Hawaii.

**Conclusions:**

From 1999 to 2020, mortality rates for adults aged ≥45 years with concurrent lung cancer and CIHD declined. The highest AAMRs were observed among men, NH Whites, individuals residing in the Midwest, and non-metropolitan populations. This highlights the need for a more comprehensive and tailored approach to managing these patients moving forward.

## 1 Introduction

Lung cancer, classified as non-small-cell lung cancer (NSCLC) and small-cell lung cancer (SCLC), is the leading cause of cancer-related mortality across the globe and the second-most frequently diagnosed cancer after breast cancer in women and prostate cancer in men [1]. GLOBOCAN 2020 estimates suggested approximately 2.2 million new lung cancer diagnoses and 1.8 million lung cancer deaths in the year 2020 [2]. Comorbidities can alter the cancer risk, diagnosis, disease evolution, choice of treatment, and survival outcomes [3]. Frequently encountered comorbidities in lung cancer patients include hypertension, coronary artery disease, and diabetes mellitus, which may affect the prognosis and survival. Moreover, there is a strong association between mortality and heart failure in lung cancer patients [4,5].

There is a remarkable increase in the incidence of cardiovascular disorders in cancer patients, which can be attributed to the overlapping of risk factors and cardiotoxic effects of cancer treatment [4,6]. The prevalent risk factors include obesity, air pollution, and tobacco smoking, which may accelerate both lung cancer and cardiovascular disorders. Besides, anti-tumor treatments such as cisplatin result in the development of arrhythmias, atrial fibrillation, and bradycardia; trastuzumab leads to left ventricular dysfunction; immune checkpoint inhibitors are associated with fatal myocarditis [7,8]. Zhang et al. further demonstrated a bidirectional association between NSCLC and cardiovascular disorders. The study findings suggested that lung cancer patients had high genetic risk and incidence of cardiovascular disorders, however, there is no causal relationship between lung cancer and cardiovascular co-morbidities [9].

The high comorbid disease burden in the elderly and the association of multiple chronic conditions with prognosis and survival outcomes in lung cancer patients impose a significant health burden [10]. This study aims to comprehensively examine trends and regional disparities in chronic ischemic heart disease and lung cancer mortality among American adults. By analyzing mortality data over a specified timeframe, the authors seek to identify patterns, temporal changes, and geographic variations in disease burden. Education and a thorough understanding of these trends allude to the implementation of targeted public health measures, effective allocation of resources, and ultimately reduction in the morbidity and mortality associated with lung cancer and chronic ischemic heart disease.

## 2 Methods

### 2.1 Study setting and population

This cross-sectional investigation explores CIHD and LC-related deaths occurring within the United States using the CDC-WONDER (Centers for Disease Control and Prevention Wide-ranging OnLine Data for Epidemiologic Research) database. The Multiple Cause-of-Death Public Use record death certificates were studied to determine CIHD and LC as either underlying or contributing causes of death on nationwide death certificates [11]. Previous literature has employed use of this database to analyze trends in both cardiovascular and LC-related mortality [[12], [13], [14]]. Death records were identified using codes from the International Classification of Diseases and Related Health Problems −10th Revision (ICD-10). The ICD-10 codes used for CIHD were I25.0 - I25.6, and I25.8 - I25.9 [15] while the ones used to verify LC were C34.0 - C34.3, and C34.8 - C34.9 [16]. Adults aged 45 or older were included in the analysis to focus on the demographic most burdened with CIHD and lung cancer and limit age-related confounders [[17], [18], [19]]. This study was exempt from local Institutional Review Board approval as the death records used were deidentified publicly available data from the CDC WONDER database which follows the STROBE (Strengthening the Reporting of Observational Studies in Epidemiology) guidelines for reporting.

### 2.2 Data extraction

Data for CIHD and LC-related deaths, population sizes, year, location of death (including medical facilities [outpatient, emergency room, inpatient, death on arrival, or status unknown], home, hospice, and nursing home/long-term facility), demographics (sex, race/ethnicity, and age), urban-rural classification, region and states were obtained. Race/ethnicities were defined as non-Hispanic (NH) White, NH Black or African American, Hispanic, or Latino, NH American Indian or Alaskan Native, and NH Asian or Pacific Islander. This classification was also used by previous CDC WONDER database studies [20]. The National Center for Health Statistics Urban-Rural Classification Scheme was used to assess the population by urban (large metropolitan area [population ≥1 million], medium/small metropolitan area [population 50,000–999,999]), and rural (non-metropolitan area [population <50,000]) counties per the 2013 U S. Census classification [21]. Regions were classified into North, Midwest, South, and West, based on definitions provided by the U.S. Census Bureau definitions [22].

### 2.3 Statistical analysis

To analyze country-wide trends in CIHD and LC-related mortality from 1999 to 2020, we calculated crude and age-adjusted mortality rates (AAMRs) per 100,000 population by year, sex, race/ethnicity, state, and urban-rural status with 95 % CIs. Crude mortality rates were determined by dividing the number of CIHD and LC-related deaths by the corresponding US population of that year. AAMRs were calculated by standardizing CIHD and LC-related deaths to the year 2000 U S. population [22]. The Joinpoint Regression Program (Joinpoint V 5.0.2, National Cancer Institute) was used to determine the annual percent change (APC) with 95 % CI in AAMR [23]. This software identifies significant variations in age-adjusted mortality rates over time by fitting log-linear regression models where temporal shifts occurred. APCs were considered increasing or decreasing if the slope describing the change in mortality was significantly different from zero using 2‐tailed *t*-testing. Statistical significance was set at P < 0.05.

## 3 Results

Between 1999 and 2020, 214,785 deaths in patients aged 45 to 85+ years were attributable to CIHD and Lung cancer (Supplementary Table 1). The overall AAMR for all age groups was revealed to be 8.4 (95 % CI: 8.3–8.4). 37.15 % of deaths occurred at medical facilities, 16.27 % at nursing homes, 5.30 % at hospices, 36.79 % at home and 4.27 % at other places that could not be included in the aforementioned categories, with the place of death of the remaining 464 patients remaining unknown. (Supplementary Table 2). Demographical data is Table 1.

### 3.1 Annual trends for chronic ischemic heart disease and lung cancer-related age-adjusted mortality rates

**Fig. 1:**
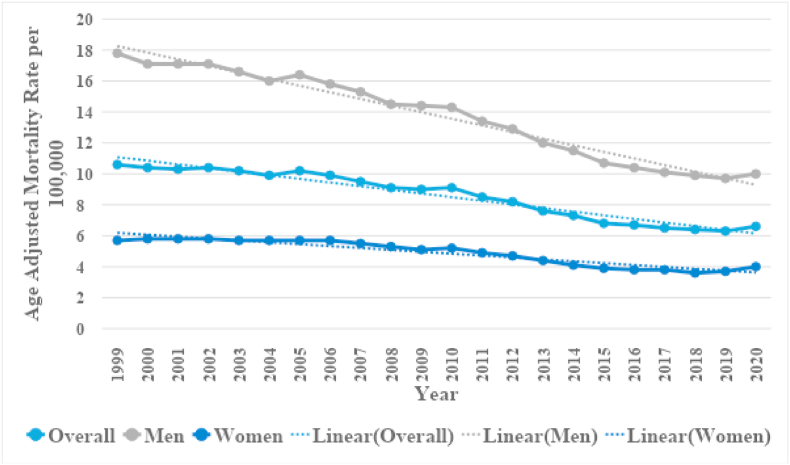
Overall and sex-stratified chronic ischemic heart disease and lung cancer-related AAMRs per 100,000 in the United States, 1999 to 2020.

In patients with CIHD with concomitant lung cancer overall AAMRs remained stable between 10.6 (95 % CI: 10.4–10.8) to 10.2 (95 % CI: 10–10.3) from 1999 to 2005 (APC: 0.8352; 95 % CI: −1.9139 to 1.5413). Over the next twelve years, a significant decrease in mortality rates was evident, with rates falling to 9.1 (95 % CI: 8.9–9.3) in 2010 (APC: −2.3654∗; 95 % CI: −5.5832 to −0.6062) and 6.5 (95 % CI: 6.4–6.7) in 2017 (APC: −4.7159∗; 95 % CI: −7.611 to −3.6). A nonsignificant uptick to 6.6 (95 % CI: 6.5–6.7) was also observed at the end of the study period in 2020 (APC: 0.8579; 95 % CI: −2.1682 to 5.2231). (Fig. 1, Supplementary Tables 3 and 4).

### 3.2 Chronic ischemic heart disease and lung cancer-related age-adjusted mortality rates stratified by sex

Deaths were distributed as 67.1 % male and 32.9 % female (Table 1). Men when compared with women had nearly triple the AAMRs throughout the study period (total AAMR men: 13.3; 95 % CI: 13.3–13.4; total AAMR women: 4.8; 95 % CI: 4.7–4.8), with rates decreasing significantly from 17.8 (95 % CI:17.3–18.2) in 1999 to 14.3 (95 % CI: 14–14.7) in 2010 (APC: −1.9901∗; 95 %: −2.3437 to −1.5586). This was succeeded by a steep decline to 10.4 (95 % CI: 10.1–10.6) in 2016 (APC: −5.4604∗; 95 % CI: −7.5203 to −4.5905). From, this point forward AAMRs remained stable reaching 10 (95 % CI: 9.8–10.3) in 2020 (APC: −1.0608; 95 % CI: −2.3142 to 1.5731). Contrastingly females showed little variation in rates between 1999 and 2011 with insignificant changes from 5.7 (95 % CI: 5.5–5.9) in 1999 to 5.7 (95 % CI: 5.5–5.9) in 2006 (APC: −0.2854; 95 % CI: −1.4654 to 0.8121) to 4.9 (95 % CI: 4.8–5.1) in 2011 (APC: −2.7531; 95 % CI: −3.478 to 0.6677). This was followed by a trend of decreasing AAMRS similar to the men reaching 3.9 (95 % CI: 3.7–4) in 2015 (APC: −5.6560∗; 95 % CI: −7.0863 to −2.3416) and 3.6 (95 % CI: 3.5–3.8) in 2018 (APC: −2.5532∗; 95 % CI: −6.1645 to −1.3115). Notably, in the tail end of the study women showed a significant increase in mortality rates inflating to 4 (95 % CI: 3.9–4.2) in 2020 (APC: 4.6547∗; 95 % CI: 1.1423 to 7.2923). (Fig. 1, Supplemental Tables 3 and 4).

### 3.3 Chronic ischemic heart disease and lung cancer-related age-adjusted mortality rates stratified by race/ethnicity

**Fig. 2:**
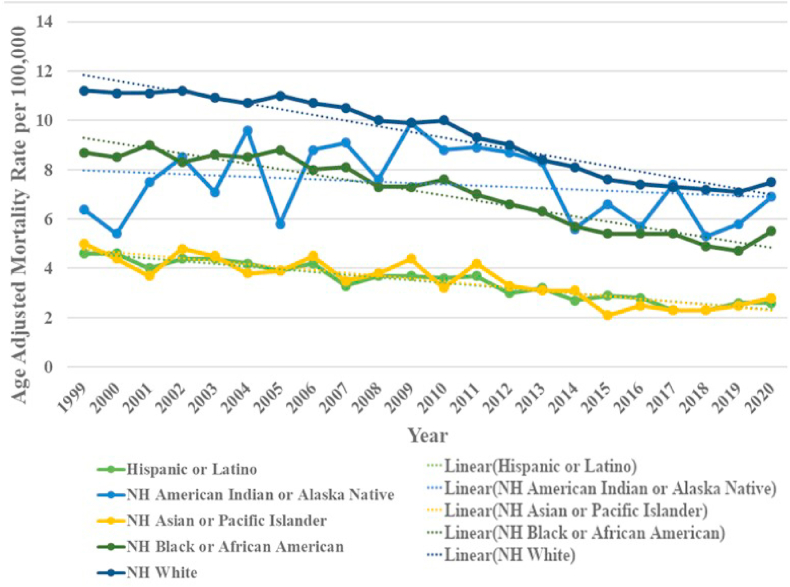
Chronic ischemic heart disease and lung cancer-related AAMRs per 100,000 stratified by race in the United States, 1999 to 2020.

When stratified by race NH Whites accounted for the majority of deaths at 88.3 % whereas NH American Indians or Alaska Natives represented 0.5 % (Table 1). AAMRs were highest in NH Whites followed by NH American Indian or Alaska Natives, NH Black or African Americans and Hispanic or Latinos with NH Asian or Pacific Islander having the lowest rates. [total AAMR NH White: 9.3 (95 % CI: 9.2–9.3); NH American Indian or Alaska Natives: 7.3 (95 % CI: 6.8–7.8); NH Black or African Americans: 6.8 (95 % CI: 6.7–6.9); Hispanic or Latino: 3.3 (95 % CI: 3.2–3.3); NH Asian or Pacific Islander: 3.2 (95 % CI: 3.1–3.4)]. To summarize, NH whites, NH Black or African Americans and Hispanics or Latinos all saw an unchanging trajectory of AAMRS from 1999 till 2005, 2005 and 2011 respectively. This was followed by a period of significant decrease in mortality till 2010 and an even greater decline till 2016 for NH whites with reducing rates being evident till 2018 for NH Black or African Americans and Hispanic or Latinos. Subsequently, all three races saw a nonsignificant increase in mortality till the end of the study period. NH American Indians or Alaska Natives saw a significant increase in the first decade of the study period, 1999–2009, (APC: 3.2519∗; 95 %: 0.1045 to 21.2117) followed by a sharp decrease till the end (APC: −4.1358∗; 95 %: −11.3373 to −1.7709). NH Asians or Pacific Islanders showed a significant decrease throughout (APC: −3.3919∗; 95 % CI: −4.374 to −2.3192). (Fig. 2, Supplemental Tables 3 and 5).

**Table 1:** Demographic Characteristics of Deaths due to Chronic Ischemic Heart disease and Lung Cancer in Adults in the United States, 1999 to 2020.

Variable	Chronic Ischemic Heart disease and Lung Cancer Deaths n (%)	AAMRs (95 % CI) per 100,000
**Overall Population**	214785 (100)	8.4 (8.3–8.4)
**Sex**		
Male	144167 (67.1)	13.3 (13.3–13.4)
Female	70618 (32.9)	4.8 (4.7–4.8)
**Census Region**		
Northeast	44163 (20.6)	8.8 (8.7–8.9)
Midwest	54908 (25.6)	9.6 (9.5–9.7)
South	78946 (36.8)	8.4 (8.3–8.5)
West	36768 (17.1)	6.8 (6.7–6.9)
**Race/Ethnicity**		
NH American Indian or Alaska Native	972 (0.5)	7.3 (6.8–7.8)
NH Asian or Pacific Islander	2954 (1.4)	3.2 (3.1–3.4)
NH Black or African American	15560 (7.3)	6.8 (6.7–6.9)
NH White	189153 (88.3)	9.3 (9.2–9.3)
Hispanic or Latino	5687 (2.7)	3.3 (3.2–3.3)
**Urbanization**		
Metropolitan	167470 (78.0)	8 (8–8)
Nonmetropolitan	47315 (22.0)	10.3 (10.2–10.4)
**Place of Death**^a^		
Medical Facility	79794 (37.2)	–
Decedent's Home	79016 (36.8)	–
Hospice Facility	11392 (5.3)	–
Nursing Home/Long-term Care Facility	34956 (16.3)	–
Others	9163 (4.3)	–
Unknown	464 (0.1)	–

NH: Non-Hispanic.

Note.

a Age Adjusted Mortality Rates (AAMRs) are not applicable for Place of Death.

### 3.4 Chronic ischemic heart disease and lung cancer-related age-adjusted mortality rates stratified by geographic region

**Fig. 3:**
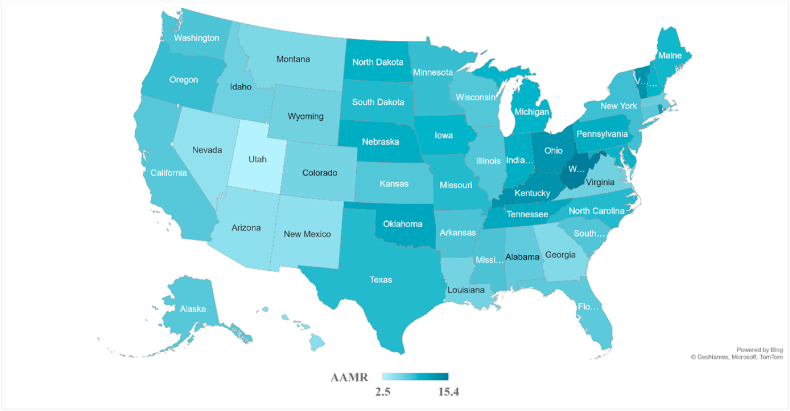
Chronic ischemic heart disease and lung cancer-related AAMRs per 100,000 stratified by state in the United States, 1999 to 2020.

**Fig. 4:**
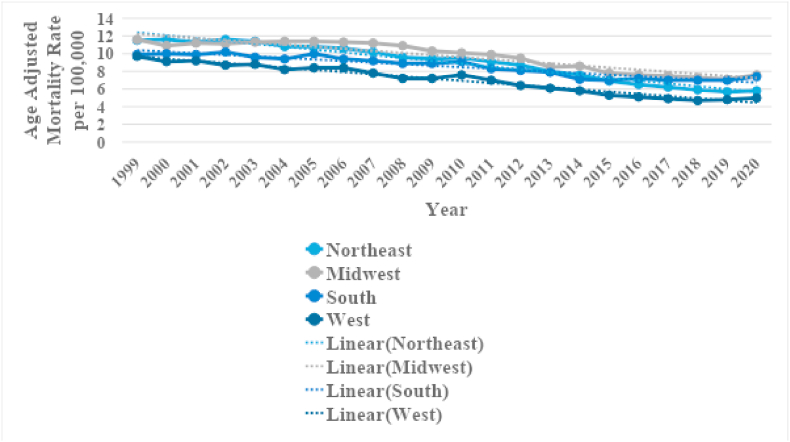
Chronic ischemic heart disease and lung cancer-related AAMRs per 100,000 stratified by census region in the United States, 1999 to 2020.

Assessing AAMRs by state AAMRs ranged from 2.5 (95 % CI: 2.3–2.8) in Utah to 15.4 (95 % CI: 14.8–15.9) in West Virginia with rates in the top 90th percentile (Rhode Island, Ohio, Vermont, Kentucky and West Virginia) being nearly triple than those in the lower 10th percentile (Utah, Nevada, Arizona, New Mexico and Hawaii). (Fig. 3 Supplemental Table 6). 36.8 % of deaths due to CIHD and concomitant lung cancer took place in the South, 25.6 % in the Midwest, 20.6 % in the Northeast and 17.1 % in the West (Table 1). According to census regions the AAMRs were highest in the Midwest (AAMR: 9.6; 95 % CI: 9.5–9.7) followed by the Northeast (AAMR: 8.8; 95 % CI: 8.7–8.9), South (AAMR: 8.4; 95 % CI: 8.3–8.5) and the West (AAMR: 6.8; 95 % CI: 6.7–6.9) (Fig. 4, Supplemental Table 7).

**Fig. 5:**
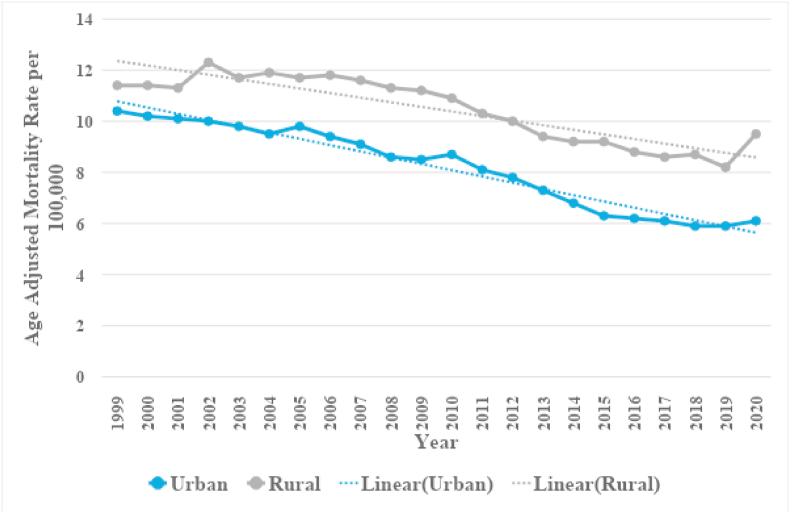
Chronic ischemic heart disease and lung cancer-related AAMRs per 100,000 stratified by urban-rural status in the United States, 1999 to 2020.

When analyzing urbanization status the majority of deaths (78.0 %) took place in metropolitan areas with non-metropolitan areas accounting for 22.0 % (Table 1). From 1999 to 2020 non-metropolitan areas had a consistently higher AAMR as compared to metropolitan areas with overall AAMRS being 10.3 (95 % CI: 10.2–10.4) and 8 (95 % CI: 8-8) respectively. Initially, nonmetropolitan area death rates remained stable between 1999 and 2007 (APC: 0.3016; 95 % CI: −0.4432 to 1.3679) contrasted with a significant decrease between 1999 and 2010 (APC: −1.7746∗; 95 % CI: −2.1122 to −1.386) evident in metropolitan areas. Both groups showed decreases in mortality from CIHD and Lung cancer till 2018 (APC: −3.1494∗; 95%CI: −4.1182 to −2.7404) and 2016 (APC: −5.6180∗; 95%CI: −8.1638 to −4.6465) respectively. By 2020 nonmetropolitan areas showed a stark significant increase in AAMRs (APC: 5.8093∗; 95 % CI: 0.1896 to 8.9844) compared to AAMRs for metropolitan areas which showed little change (APC: −0.4558; 95 % CI: −2.5751 to 4.2467). (Fig. 5, Supplemental Tables 3 and 8).

### 3.5 Chronic ischemic heart disease and lung cancer-related age-adjusted mortality rates in patients older than 65 years

In patients above 65 years old CIHD and concomitant lung cancer were responsible for 187058 (87.1 %) deaths. Overall AAMR was substantially higher in this age group at 20.54 (95 % CI: 20.45–20.63). AAMRs were found to decrease significantly from 25.5 (95 % CI: 24.97 to 26.03) in 1999 to 22.52 (95 % CI: 22.05 to 22.99) in 2010 (APC: −1.3462∗; 95 % CI: −1.7394 to −0.7852) to 16.02 (15.17–16.87) in 2016 (APC: −5.3943∗; 95 % CI: −8.281 to −4.4161). Toward the end of the study, AAMRs stabilized to 16.24 (95 % CI: 15.89 to 16.58) by 2020 (APC: −0.3337; 95 % CI: −2.6457 to 4.85). (Supplemental Tables 9, 10 and 13, Supplementary Fig. 1).

Both males and females above 65 years of age had notably higher AAMRs when compared to adults over 45. Similar findings of males having a higher total AAMR as compared to their female counterparts was noted. (total AAMR male: 32.97; 95 % CI: 32.79–33.16 vs female: 11.78; 95 % CI: 11.69–11.87). In males AAMRs decreased from 1999 to 2010 (APC: −1.8597∗; 95 % CI: −2.3047 to −1.271) and from 2010-16 (APC: −5.5870∗; 95 % CI: −8.4914 to −4.5191). In the last four years of the study, no significant variations were seen (APC: −0.8529; 95 % CI: −2.7859 to 3.5951). In females AAMRs remained stable in the initial years till 2005 (APC: 0.1875; 95 % CI: −0.6871 to 2.6805) and dropped significant between 2005 and 2010 (APC: 2.0212∗; 95 % CI: −4.2681 to −0.9449) and then again between 2010 and 2018 (APC: 4.8931∗; 95 % CI: −6.146 to −4.3787) Similar to trends seen in females >45 years a significant increase in mortality was seen in the later years of the study till 2020 (APC: 6.8228∗; 95 % CI: 2.6551 to 9.5414). (Supplemental Tables 10 and 13, Supplementary Fig. 1).

When analyzing race-related mortality NH Whites had the highest AAMRs (AAMR: 22.58; 95 % CI:22.47–22.69) followed by NH American Indian or Alaska Natives (AAMR: 17.3; 95 % CI: 16.03–18.57) NH Black or African Americans (AAMR: 15.98; 95 % CI: 15.7–16.27) NH Asian or Pacific Islander (AAMR: 8.35; 95 % CI: 8.03–8.67) and Hispanic or Latinos (AAMR: 8.32; 95 % CI: 8.09–8.56). AAMRs for NH Whites followed overall trends decreasing significantly from 1999 to 2010 (APC: −1.0384∗; 95 % CI: −1.5313 to −0.3916) till 2016 APC: −5.2007∗; 95 % CI: −8.5806 to −3.9771) and showed little variation from this point forward stabilizing by in 2020. (APC: 0.0081; 95 % CI: −2.3768 to 5.2303). Hispanic or Latinos showed a significant decrease throughout the study period. (APC: −3.1362∗; 95 % CI: −3.6886 to −2.5141). Trends for NH Black or African Americans were identical to trends observed for patients >45 years in this subgroup with stable trends observed till 2005 (APC: 0.3671; 95 % CI: −2.4055 to 4.6193) followed by a decrease till 2018 (APC: −4.1271∗; 95 % CI: −8.7494 to −1.0908) after which another period of stability till 2020 (APC: 4.1912; 95 % CI: −3.7709 to 9.0107) was noted. In NH American Indians or Alaskan natives aged 65 and above trends of significant increase till 2009, (APC: 3.7030∗; 95 % CI: 0.5491 to 17.9935) followed by significant decrease (APC: −3.9377∗; 95 % CI: −10.312 to −1.6338) were noted. Similarly, results for NH Asian or Pacific Islanders also bore a resemblance to the 45 and above age group showing a significant decrease throughout. (APC: −3.4247∗; 95 % CI: −4.3927 to −2.3074). (Supplemental Tables 10 and 13, Supplementary Fig. 2).

Stratifying by geography trends analogous to the ones observed in patients >45 years were seen, with the Midwest region having the highest mortality rates (AAMR: 23.3; 95 % CI: 23.09–23.51), followed by the Northeast (AAMR: 21.86; 95 % CI: 21.64–22.07), the South (AAMR: 20.19; 95 % CI: 20.04–20.34), and lastly, the West (AAMR: 17.11; 95 % CI: 16.93–17.3). Likewise, state-level variations were also similar with Rhode Island, Ohio, Vermont, Kentucky, and West Virginia being states in the top 90th percentile. (Supplemental Tables 11–13, Supplementary Fig. 3).

Looking at urbanization status, nonmetropolitan areas (AAMR: 4.56; 95 % CI: 24.32–24.8) had higher rates than metropolitan ones (AAMR: 19.68; 95 % CI:19.58–19.78) with the rural-urban gap widening in older patients. Metropolitan AAMRs for patients above 65 years of age showed significant decreases from 1999 to 2010 (APC: −1.5606∗; 95 % CI: −1.9296 to −1.1052) and from 2010 to 2016 (APC: −5.7398∗; 95 % CI: −8.023 to −4.7922). Changes till 2020 were insignificant (APC: −0.3945; 95 % CI: −2.1287 to 3.2962). Mortality rates remained stable till 2007 (APC: 0.4694; 95 % CI: −0.4181 to 1.6495) after which a steep decrease was observed till 2018 (APC: −3.1755∗; 95 % CI: −4.941 to −2.6528) for patients residing in nonmetropolitan areas. Additionally, rates remained stable till the end of the study period (APC: 4.0938; 95 % CI: −1.6296 to 7.2354). (Supplemental Tables 11 and 13, Supplementary Fig. 4).

## 4 Discussion

In this retrospective analysis of the CDC WONDER database, we report several key findings. First, overall mortality associated with CIHD, and lung cancer decreased throughout the study period. Second, males were reported to have persistently higher mortality rates than females. Third, considerable variations in mortality were observed among different racial subgroups, with NH White adults exhibiting the highest AAMRs. Fourth, disparities in AAMRs were evident across different census regions, with non-metropolitan areas reporting constantly higher AAMRs. Finally, states in the upper 90th percentile (Rhode Island, Ohio, Vermont, Kentucky, and West Virginia) displayed AAMRs nearly three times those in the lower 10th percentile. These trends have significant implications for targeted healthcare improvements in reducing the overall mortality attributed to CIHD and lung cancer.

Although global incidence and mortality rates have consistently increased, we observed an overall decrease in mortality associated with CIHD and lung cancer, which is mirrored by the improved 5-year survival rates for lung cancer and ischemic heart disease mortality in the last two decades in the United States [[24], [25], [26], [27], [28], [29]]. This can be attributed to increased screening rates and more advanced screening techniques that help with the early diagnosis and treatment of lung cancer [30]. A 2020 report claims that the five-year survival rate for lung cancer is 59 % when diagnosed early, and early diagnosis rates have increased by 33 % in the last five years [31]. The use of low dose computed tomography for lung cancer screening has increased from 3.3 % in 2010 to 5 % in 2018, allowing early diagnosis of lung cancer [32,33]. Advanced radical radiotherapy techniques, such as stereotactic ablative radiotherapy (SABR) and percutaneous radiofrequency ablation (RFA) under CT guidance, can also be associated with reduced mortality in lung cancer, as 2-year survival rates were shown to be 70 % with SABR vs. 53 % with conventional radiotherapy [34]. Advanced and less invasive surgical techniques, such as video-assisted thoracoscopic surgery (VATS) for lung resections, have also increased survival, as evident in a meta-analysis showing 5-year survival following VATS lobectomy for early-stage lung cancer to be 80.1 % vs. 65.6 % for open lobectomy [35]. Better tolerated, more targeted, and individually tailored systemic therapies for NSCLC, including epidermal growth factor receptor (EGFR) inhibitors, gefitinib, erlotinib, and afatinib; the anaplastic lymphoma kinase (ALK) inhibitor, crizotinib; immune checkpoint inhibitors, pembrolizumab, nivolumab, and atezolizumab; and the anti-vascular endothelial growth factor receptor monoclonal antibody bevacizumab, have also revolutionized the treatment, reducing mortality [36,37].

Advanced diagnostic and therapeutic technologies, such as laboratories for coronary angiograms and angioplasties as well as easy access to healthcare, have improved mortality due to ischemic heart disease [38]. Advancements in coronary imaging, such as computed tomography angiography and cardiac magnetic resonance, and revascularization strategies, such as coronary artery bypass grafting and percutaneous coronary intervention, have vastly improved mortality outcomes [39,40]. Therapies once considered experimental, such as autologous bone marrow stem cell transplantation and microRNA modulation for the de-repression of survival and pro-angiogenic genes, are also now reducing CIHD mortality [41,42]. Improvements in the management of other comorbidities, such as hyperlipidemia, hypertension, diabetes mellitus, and chronic kidney disease, have also led to improved mortality [[43], [44], [45], [46]]. Smoking induces oxidative stress, vascular inflammation, platelet coagulation, vascular dysfunction, and impairs the serum lipid profile, which is linked to worsening mortality outcomes in chronic obstructive pulmonary disease, idiopathic pulmonary fibrosis, atherosclerosis, CIHD, and lung cancer [[47], [48], [49]]. Strict tobacco control programs and policies, including mass media campaigns, excise taxes on tobacco, smoke-free air policies, and restricted access to youth in recent years, have led to the control of the tobacco epidemic in the United States, showing positive associations with decreased mortality due to CIHD and lung cancer [[50], [51], [52]].

Males showed higher AAMRs throughout the study period. Various studies have shown females are less prone to cardiovascular diseases, lowering the revascularization intervention rates, and many studies have focused on the cardioprotective role of estrogen [[53], [54], [55]]. The disparity could also be explained on the basis of occupation, as males were not only more sensitive but also more exposed to occupational lung diseases, leading to increased carcinogenesis [[56], [57], [58]]. Initially, males had three times the AAMR than females; however, by the end of the study period, the difference narrowed to the point where males had just 2.5 times the female AAMR, and a significant increase was noted in the female AAMRs from 2018 to 2020, while the male AAMRs decreased. This disparity could be because of the tobacco epidemic hitting females at the time when it started to decrease in males, which is evident in studies stating that declines began later and have been slower in women than in men because women took up cigarette smoking in large numbers later and were slower to quit, including upturns in smoking prevalence [[59], [60], [61]].

When stratified by race/ethnicity, significant disparities in CIHD and lung-cancer-related mortality were observed. Although studies have shown that mortality rates among NH Blacks from coronary heart disease are almost two-fold higher than those among NH Whites and that there is lower adherence to lung cancer screening linked with poorer prognosis, our analysis shows that the highest mortality rates are observed in NH Whites [[62], [63], [64], [65]]. This disparity could be explained on the genetic level as studies analyzing mutation frequencies associated with epidermal growth factor receptor (EGFR) have reported lower mutation frequencies in black individuals as compared to white individuals [[66], [67], [68]]. Furthermore, higher high-density lipoprotein cholesterol (HDL-C) levels, which are considered cardioprotective, were observed in NH Blacks, and lower 25(OH) vitamin D concentration, which was associated with a greater risk of coronary heart disease incidence, was observed in NH Whites, which aligns with our results [69,70]. We observed that AAMRs for NH American Indians and Alaska Natives showed a significant increase in the first half of our study period, making the mortality rates even higher than those of NH Blacks. This aligns with results from another study that reported lower survival rates for NH-American Indians or Alaska Natives, and greater smoking prevalence increases lung cancer mortality [71]. Hispanic or Latinos and NH Asians or Pacific Islanders were associated with lower AAMRs, with an overall significant decreasing trend, which is in line with results from other mortality analysis studies [[72], [73], [74]].

Geographical disparities throughout the study duration were observed, with the highest AAMRs observed in the Midwestern region, which could be due to a higher prevalence of smoking and lower cessation rates associated with the slowest decrease in lung cancer in the Midwest [[75], [76], [77]]. Metropolitan areas showed consistently lower AAMRs, with a decreasing trend, whereas AAMRs for non-metropolitan areas decreased until 2018, after which a significant increase was observed, widening the mortality gap. Generally, rural areas demonstrate delayed diagnosis and treatment, which may be related to a lack of resources, infrastructure, and transportation [78]. Furthermore, a higher proportion of the aging population, lower socioeconomic status, and lower literacy rates are associated with increased mortality [79]. The disparities in healthcare are also evident in the states reflecting the prevalence of risk factors as well as prevention and early detection practices. Our findings highlight the necessity of conducting extensive population-based studies within these regions to identify key factors contributing to the observed disparity.

Ischemic heart disease is the leading global cause of death and is expected to remain the leading cause of death by 2050 at the global level and increase in prevalence by 31.1 % by the year 2060 [80,81]. For lung cancer, global age-standardized incidence rates were predicted to decrease by 23 % among males and increase by 2 % among females by 2035 [82]. More research is required to explain and narrow the disparities in mortality data for gender, racial groups, and geographical regions. The findings underscore the need to intensify efforts aimed at early diagnosis and treatment, evaluation and control of risk factors and comorbid conditions, assessment of inequalities in access to healthcare, implementation of culturally customized interventions, and extensive population-based studies to identify factors contributing to the observed disparity.

Our study results should be interpreted with some limitations in mind. The use of ICD-10 codes for diagnosis and reliance on death certificates to ascertain the cause of death can lead to misdiagnosis. The classification of the various subtypes of the included diseases was not explained. Data regarding competing risk of death could not be assessed due to search limitations of the WONDER database. Detailed laboratory work for each patient is not provided. The database cannot offer details on the various treatments and procedures that may have been performed. The final place of death might not be the location from where the patient belonged, therefore skewing geographical data.

## 5 Conclusion

In conclusion, our analysis of mortality due to CIHD and lung cancer, which includes data from the United States, shows an overall decrease in mortality. Males have a higher mortality rate than females but the gap in mortality rates is narrowing. Disparities were observed based on race/ethnicity and geographical region, with the highest AAMRs observed in NH Whites and the Midwestern region. Targeted therapy and newer policies are required to reduce the mortality rate and disease burden of CIHD and lung cancer. Further studies are required to establish the patterns observed in our study.

## CRediT authorship contribution statement

**Eman Ali:** Writing – original draft, Methodology, Formal analysis, Data curation, Conceptualization. **Hafsah Alim Ur Rahman:** Data curation, Formal analysis, Writing – original draft. **Usama Hussain Kamal:** Conceptualization, Data curation, Investigation. **Muhammad Ahmed Ali Fahim:** Data curation, Formal analysis, Writing – original draft. **Madiha Salman:** Conceptualization, Resources, Writing – original draft. **Afia Salman:** Data curation, Software, Writing – original draft. **Hamza Nawaz Khan:** Investigation, Methodology, Writing – original draft. **Farah Yasmin:** Project administration, Supervision, Writing – review & editing. **Chmsalddin Alkhas:** Formal analysis, Software, Writing – review & editing. **Afsana Ansari Shaik:** Investigation, Methodology, Writing – review & editing. **Muhammad Sohaib Asghar:** Writing – review & editing, Visualization, Validation, Supervision, Project administration. **M. Chadi Alraies:** Funding acquisition, Project administration, Writing – review & editing.

## Declaration of competing interest

The authors declare that they have no known competing financial interests or personal relationships that could have appeared to influence the work reported in this paper.
