# Supplementary material for: Trends and regional variations in chronic ischemic heart disease and lung cancer-related mortality among American adults: Insights from retrospective CDC wonder analysis
Source: Int J Cardiol Cardiovasc Risk Prev. 2025 Feb 14;24:200377. doi: 10.1016/j.ijcrp.2025.200377 (PMC11875809; doi:10.1016/j.ijcrp.2025.200377)
Supplement: Multimedia component 1 [file mmc1.docx]

| **Year** | **Overall** | **Women** | **Men** | **NH White** | **NH Black or African American** | **NH Asian or Pacific Islander** | **NH American Indian or Alaska Native** | **Hispanic or Latino** | **Population** |
| --- | --- | --- | --- | --- | --- | --- | --- | --- | --- |
| 1999 | 10095 | 3234 | 6861 | 9066 | 686 | 100 | 25 | 195 | 95153686 |
| 2000 | 10011 | 3273 | 6738 | 8970 | 685 | 100 | 22 | 211 | 96944389 |
| 2001 | 10128 | 3313 | 6815 | 9054 | 730 | 84 | 33 | 192 | 99781854 |
| 2002 | 10311 | 3392 | 6919 | 9215 | 688 | 118 | 39 | 228 | 102217733 |
| 2003 | 10251 | 3370 | 6881 | 9114 | 726 | 121 | 30 | 232 | 104692428 |
| 2004 | 10144 | 3427 | 6717 | 9008 | 731 | 106 | 43 | 237 | 107138553 |
| 2005 | 10531 | 3461 | 7070 | 9369 | 770 | 115 | 29 | 236 | 109787199 |
| 2006 | 10400 | 3480 | 6920 | 9217 | 714 | 141 | 46 | 263 | 112380379 |
| 2007 | 10240 | 3389 | 6851 | 9090 | 760 | 119 | 49 | 213 | 114894084 |
| 2008 | 9961 | 3340 | 6621 | 8828 | 689 | 131 | 40 | 257 | 117395131 |
| 2009 | 10031 | 3293 | 6738 | 8831 | 715 | 155 | 54 | 267 | 119895863 |
| 2010 | 10260 | 3419 | 6841 | 9046 | 755 | 125 | 50 | 270 | 121757429 |
| 2011 | 9902 | 3301 | 6601 | 8626 | 728 | 174 | 52 | 306 | 124174484 |
| 2012 | 9761 | 3223 | 6538 | 8586 | 702 | 145 | 54 | 253 | 126000296 |
| 2013 | 9369 | 3059 | 6310 | 8156 | 706 | 146 | 52 | 289 | 127788037 |
| 2014 | 9140 | 2905 | 6235 | 8006 | 655 | 156 | 42 | 258 | 129779643 |
| 2015 | 8805 | 2834 | 5971 | 7657 | 651 | 118 | 45 | 301 | 131826832 |
| 2016 | 8816 | 2879 | 5937 | 7635 | 674 | 143 | 47 | 293 | 133494018 |
| 2017 | 8855 | 2879 | 5976 | 7661 | 702 | 141 | 60 | 263 | 135229289 |
| 2018 | 8929 | 2873 | 6056 | 7800 | 649 | 145 | 46 | 266 | 136335528 |
| 2019 | 9062 | 2978 | 6084 | 7838 | 659 | 174 | 49 | 319 | 137381702 |
| 2020 | 9783 | 3296 | 6487 | 8380 | 785 | 197 | 65 | 338 | 138429175 |
| **Total** | 214785 | 70618 | 144167 | 189153 | 15560 | 2954 | 972 | 5687 | 2622477732 |

Supplementary Table 1: Supplemental Table 1 Lung Cancer and Ischemic Heart Disease related Deaths, Stratified by Sex and Race, in Adults in the United States, 1999 to 2020

| **Deaths** | | | | | | |
| --- | --- | --- | --- | --- | --- | --- |
| **Year** | **Medical Facility** | **Nursing Home** | **Hospices** | **Home** | **Other** | **Unknown** |
|  |  |  |  |  |  |  |
| 1999 | 4837 | 1730 | - | 3183 | 342 | - |
| 2000 | 4715 | 1767 | - | 3144 | 384 | - |
| 2001 | 4690 | 1771 | - | 3262 | 401 | - |
| 2002 | 4650 | 1789 | - | 3377 | 493 | - |
| 2003 | 4483 | 1776 | 32 | 3454 | 484 | 22 |
| 2004 | 4338 | 1783 | 60 | 3414 | 526 | 23 |
| 2005 | 4296 | 1841 | 205 | 3652 | 506 | 31 |
| 2006 | 4201 | 1874 | 292 | 3520 | 475 | 38 |
| 2007 | 4073 | 1792 | 390 | 3550 | 422 | 13 |
| 2008 | 3814 | 1746 | 448 | 3421 | 422 | 110 |
| 2009 | 3560 | 1813 | 517 | 3468 | 502 | 171 |
| 2010 | 3788 | 1637 | 617 | 3670 | 539 | - |
| 2011 | 3371 | 1681 | 667 | 3708 | 471 | - |
| 2012 | 3220 | 1537 | 776 | 3739 | 479 | 10 |
| 2013 | 3035 | 1480 | 776 | 3595 | 476 | - |
| 2014 | 2812 | 1438 | 855 | 3708 | 320 | - |
| 2015 | 2782 | 1306 | 896 | 3531 | 288 | - |
| 2016 | 2711 | 1345 | 929 | 3565 | 266 | - |
| 2017 | 2644 | 1297 | 1002 | 3622 | 288 | - |
| 2018 | 2602 | 1236 | 1000 | 3767 | 320 | - |
| 2019 | 2597 | 1206 | 1010 | 3903 | 346 | - |
| 2020 | 2575 | 1111 | 920 | 4763 | 413 | - |
| **Total** | 79794 | 34956 | 11392 | 79016 | 9163 | 464 |

Supplemental Table 2: Lung cancer and Ischemic Heart Disease related Mortality, Stratified by Place of Death in Adults in the United States, 1999 to 2020

Supplemental Table 3 Annual percent change (APC) of Lung Cancer and Ischemic Heart Disease related Age-Adjusted Mortality Rates per 100,000 in Adults in the United States, 1999 to 2020

| **Year Interval** | **APC (95% CI)** |
| --- | --- |
| **Overall** | |
| 1999-2005 | -0.8352 (-1.9139 to 1.5413) |
| 2005-2010 | -2.3654* (-5.5832 to -0.6062) |
| 2010-2017 | -4.7159* (-7.611 to -3.6) |
| 2017-2020 | 0.8579 (-2.1682 to 5.2231) |
| **Male** | |
| 1999-2010 | -1.9901* (-2.3437 to -1.5586) |
| 2010-2016 | -5.4604* (-7.5203 to -4.5905) |
| 2016-2020 | -1.0608 (-2.3142 to 1.5731) |
| **Female** | |
| 1999-2006 | -0.2854 (-1.4654 to 0.8121) |
| 2006-2011 | -2.7531 (-3.478 to 0.6677) |
| 2011-2015 | -5.6560* (-7.0863 to -2.3416) |
| 2015-2018 | -2.5532* (-6.1645 to -1.3115) |
| 2018-2020 | 4.6547* (1.1423 to 7.2923) |
| **NH White** | |
| 1999-2005 | -0.5251 (-1.1131 to 1.1749) |
| 2005-2010 | -1.9775* (-4.7759 to -1.1697) |
| 2010-2016 | -4.8506* (-6.9421 to -3.7813) |
| 2016-2020 | 0.0759 (-1.5302 to 3.5532) |
| **NH Black or African American** | |
| 1999-2005 | 0.1061 (-3.8278 to 4.867) |
| 2005-2018 | -4.218 (-9.4572 to 2.5106) |
| 2018-2020 | 3.6278 (-4.1305 to 8.301) |
| **NH American Indian or Alaska Native** | |
| 1999-2009 | 3.2519* (0.1045 to 21.2117) |
| 2009-2020 | -4.1358* (-11.3373 to -1.7709) |
| **NH Asian or Pacific Islander** | |
| 1999-2020 | -3.3919* (-4.374 to -2.3192) |
| **Hispanic or Latino** | |
| 1999-2011 | -2.2813 (-3.0756 to 0.2376) |
| 2011-2018 | -5.7122* (-11.8164 to -4.2171) |
| 2018-2020 | 7.5667 (-0.1796 to 13.6154) |
| **Nonmetropolitan areas** | |
| 1999-2007 | 0.3016 (-0.4432 to 1.3679) |
| 2007-2018 | -3.1494* (-4.1182 to -2.7404) |
| 2018-2020 | 5.8093* (0.1896 to 8.9844) |
| **Metropolitan area** | |
| 1999-2010 | -1.7746* (-2.1122 to -1.386) |
| 2010-2016 | -5.6180* (-8.1638 to -4.6465) |
| 2016-2020 | -0.4558 (-2.5751 to 4.2467) |
| **Northeast region** | |
| 1999-2002 | 0.2291 (-1.1135 to 2.5233) |
| 2002-2011 | -2.5919* (-3.1224 to -2.2359) |
| 2011-2018 | -6.3566* (-8.1856 to -5.7606) |
| 2018-2020 | 0.0274 (-3.3837 to 2.363) |
| **Midwest region** | |
| 1999-2007 | 0.1276 (-0.678 to 0.9657) |
| 2007-2018 | -4.1242* (-5.8498 to -0.5775) |
| 2018-2020 | 2.4908 (-3.7081 to 5.6823) |
| **South region** | |
| 1999-2010 | -1.2052* (-1.6381 to -0.3197) |
| 2010-2015 | -4.7374* (-7.5155 to -3.4469) |
| 2015-2020 | 0.6907 (-0.8468 to 5.1009) |
| **West region** | |
| 1999-2010 | -2.4841* (-3.0186 to -1.7815) |
| 2010-2018 | -5.5427* (-8.2668 to -4.7267) |
| 2018-2020 | 4.3094 (-1.0566 to 7.4622) |

APC = annual percent change; NH = non-Hispanic; * Indicates that the annual percentage change (APC) is significantly different from zero at α = 0.05. AAMR = age-adjusted mortality rate.

| **Age-Adjusted Rate (95% CI)** | | | |
| --- | --- | --- | --- |
| **Year** | **Male** | **Female** | **Overall** |
| 1999 | 17.8 (17.3-18.2) | 5.7 (5.5-5.9) | 10.6 (10.4-10.8) |
| 2000 | 17.1 (16.7-17.5) | 5.8 (5.6-6) | 10.4 (10.2-10.6) |
| 2001 | 17.1 (16.7-17.5) | 5.8 (5.6-6) | 10.3 (10.1-10.5) |
| 2002 | 17.1 (16.7-17.5) | 5.8 (5.6-6) | 10.4 (10.2-10.6) |
| 2003 | 16.6 (16.3-17) | 5.7 (5.5-5.9) | 10.2 (10-10.4) |
| 2004 | 16 (15.6-16.3) | 5.7 (5.5-5.9) | 9.9 (9.8-10.1) |
| 2005 | 16.4 (16-16.8) | 5.7 (5.6-5.9) | 10.2 (10-10.3) |
| 2006 | 15.8 (15.4-16.2) | 5.7 (5.5-5.9) | 9.9 (9.7-10.1) |
| 2007 | 15.3 (14.9-15.6) | 5.5 (5.3-5.7) | 9.5 (9.3-9.7) |
| 2008 | 14.5 (14.1-14.8) | 5.3 (5.1-5.5) | 9.1 (9-9.3) |
| 2009 | 14.4 (14.1-14.8) | 5.1 (5-5.3) | 9 (8.8-9.2) |
| 2010 | 14.3 (14-14.7) | 5.2 (5.1-5.4) | 9.1 (8.9-9.3) |
| 2011 | 13.4 (13.1-13.7) | 4.9 (4.8-5.1) | 8.5 (8.4-8.7) |
| 2012 | 12.9 (12.6-13.2) | 4.7 (4.6-4.9) | 8.2 (8-8.4) |
| 2013 | 12 (11.7-12.3) | 4.4 (4.2-4.5) | 7.6 (7.5-7.8) |
| 2014 | 11.5 (11.2-11.8) | 4.1 (3.9-4.2) | 7.3 (7.1-7.4) |
| 2015 | 10.7 (10.4-10.9) | 3.9 (3.7-4) | 6.8 (6.6-6.9) |
| 2016 | 10.4 (10.1-10.6) | 3.8 (3.7-4) | 6.7 (6.5-6.8) |
| 2017 | 10.1 (9.8-10.4) | 3.8 (3.6-3.9) | 6.5 (6.4-6.7) |
| 2018 | 9.9 (9.7-10.2) | 3.6 (3.5-3.8) | 6.4 (6.2-6.5) |
| 2019 | 9.7 (9.5-10) | 3.7 (3.5-3.8) | 6.3 (6.2-6.4) |
| 2020 | 10 (9.8-10.3) | 4 (3.9-4.2) | 6.6 (6.5-6.7) |
| **Total** | **13.3 (13.3-13.4)** | **4.8 (4.7-4.8)** | **8.4 (8.3-8.4)** |

Supplemental Table 4 Overall and Sex‐Stratified Lung cancer and Ischemic Heart disease-related Age-Adjusted Mortality Rates per 100,000 in Adults in the United States, 1999 to 2020

| **Age-Adjusted Rate (95% CI)** | | | | | |
| --- | --- | --- | --- | --- | --- |
| **Year** | **NH White** | **NH Black or African American** | **NH American Indian or Alaska Native** | **Hispanic or Latino** | **NH Asian or Pacific Islander** |
| 1999 | 11.2 (11-11.5) | 8.7 (8-9.4) | 6.4 (4-9.7) | 4.6 (3.9-5.2) | 5 (4-6) |
| 2000 | 11.1 (10.9-11.3) | 8.5 (7.9-9.2) | 5.4 (3.3-8.3) | 4.6 (4-5.2) | 4.4 (3.5-5.2) |
| 2001 | 11.1 (10.9-11.3) | 9 (8.3-9.6) | 7.5 (5.1-10.7) | 4 (3.5-4.6) | 3.7 (2.9-4.5) |
| 2002 | 11.2 (11-11.4) | 8.3 (7.7-9) | 8.5 (5.9-11.8) | 4.4 (3.8-5) | 4.8 (3.9-5.6) |
| 2003 | 10.9 (10.7-11.2) | 8.6 (7.9-9.2) | 7.1 (4.7-10.3) | 4.4 (3.8-5) | 4.5 (3.6-5.3) |
| 2004 | 10.7 (10.5-10.9) | 8.5 (7.9-9.1) | 9.6 (6.9-13.1) | 4.2 (3.6-4.7) | 3.8 (3-4.5) |
| 2005 | 11 (10.8-11.3) | 8.8 (8.1-9.4) | 5.8 (3.8-8.6) | 3.9 (3.4-4.4) | 3.9 (3.2-4.6) |
| 2006 | 10.7 (10.5-10.9) | 8 (7.4-8.6) | 8.8 (6.3-11.9) | 4.2 (3.7-4.7) | 4.5 (3.7-5.2) |
| 2007 | 10.5 (10.2-10.7) | 8.1 (7.5-8.6) | 9.1 (6.5-12.3) | 3.3 (2.9-3.8) | 3.5 (2.9-4.2) |
| 2008 | 10 (9.8-10.2) | 7.3 (6.7-7.8) | 7.6 (5.3-10.6) | 3.7 (3.3-4.2) | 3.8 (3.1-4.4) |
| 2009 | 9.9 (9.7-10.1) | 7.3 (6.8-7.9) | 9.9 (7.3-13.1) | 3.7 (3.2-4.1) | 4.4 (3.7-5.1) |
| 2010 | 10 (9.8-10.2) | 7.6 (7-8.1) | 8.8 (6.4-11.7) | 3.6 (3.1-4) | 3.2 (2.7-3.8) |
| 2011 | 9.3 (9.1-9.5) | 7 (6.5-7.5) | 8.9 (6.5-11.8) | 3.7 (3.3-4.1) | 4.2 (3.5-4.8) |
| 2012 | 9 (8.8-9.2) | 6.6 (6.1-7.1) | 8.7 (6.5-11.6) | 3 (2.6-3.3) | 3.3 (2.8-3.9) |
| 2013 | 8.4 (8.2-8.6) | 6.3 (5.9-6.8) | 8.3 (6.1-10.9) | 3.2 (2.8-3.5) | 3.1 (2.6-3.6) |
| 2014 | 8.1 (7.9-8.3) | 5.7 (5.3-6.2) | 5.6 (4-7.7) | 2.7 (2.4-3) | 3.1 (2.6-3.6) |
| 2015 | 7.6 (7.4-7.8) | 5.4 (5-5.8) | 6.6 (4.7-8.9) | 2.9 (2.6-3.3) | 2.1 (1.7-2.5) |
| 2016 | 7.4 (7.3-7.6) | 5.4 (5-5.9) | 5.7 (4.1-7.7) | 2.8 (2.5-3.1) | 2.5 (2.1-2.9) |
| 2017 | 7.3 (7.1-7.5) | 5.4 (5-5.8) | 7.4 (5.6-9.6) | 2.3 (2-2.6) | 2.3 (1.9-2.6) |
| 2018 | 7.2 (7.1-7.4) | 4.9 (4.5-5.2) | 5.3 (3.9-7.2) | 2.3 (2-2.6) | 2.3 (1.9-2.7) |
| 2019 | 7.1 (7-7.3) | 4.7 (4.3-5.1) | 5.8 (4.2-7.7) | 2.6 (2.3-2.9) | 2.5 (2.2-2.9) |
| 2020 | 7.5 (7.3-7.7) | 5.5 (5.1-5.9) | 6.9 (5.3-8.9) | 2.6 (2.3-2.9) | 2.8 (2.4-3.2) |
| **Total** | 9.3 (9.2-9.3) | 6.8 (6.7-6.9) | 7.3 (6.8-7.8) | 3.3 (3.2-3.3) | 3.2 (3.1-3.4) |

Supplemental Table 5: Race‐Stratified Lung cancer and Ischemic Heart disease-related Age-Adjusted Mortality Rates per 100,000 in Adults in the United States, 1999 to 2020

NH: Non-Hispanic

Supplemental Table 6 Lung cancer and Ischemic Heart Disease related Age-Adjusted Mortality Rates per 100,000, Stratified by States in Adults in the United States, 1999 to 2020

| **State** | **Age-Adjusted Rate (95% CI)** |
| --- | --- |
| Alabama | 7.1 (6.8-7.4) |
| Alaska | 7.5 (6.5-8.4) |
| Arizona | 4.7 (4.5-4.9) |
| Arkansas | 8 (7.7-8.4) |
| California | 7.5 (7.4-7.6) |
| Colorado | 6.1 (5.8-6.3) |
| Connecticut | 7.5 (7.2-7.8) |
| Delaware | 10.2 (9.5-10.9) |
| District of Columbia | 6.4 (5.6-7.1) |
| Florida | 7.3 (7.1-7.4) |
| Georgia | 5.4 (5.3-5.6) |
| Hawaii | 5.1 (4.7-5.5) |
| Idaho | 6.5 (6-6.9) |
| Illinois | 7.7 (7.5-7.8) |
| Indiana | 10.2 (9.9-10.5) |
| Iowa | 9.7 (9.4-10.1) |
| Kansas | 7.6 (7.2-7.9) |
| Kentucky | 13.2 (12.8-13.6) |
| Louisiana | 6.2 (5.9-6.4) |
| Maine | 9.3 (8.8-9.9) |
| Maryland | 9.8 (9.5-10) |
| Massachusetts | 6.9 (6.7-7.1) |
| Michigan | 9.6 (9.4-9.8) |
| Minnesota | 8.6 (8.3-8.9) |
| Mississippi | 8 (7.6-8.3) |
| Missouri | 9 (8.7-9.3) |
| Montana | 5.7 (5.2-6.2) |
| Nebraska | 10.3 (9.8-10.8) |
| Nevada | 4.6 (4.3-4.9) |
| New Hampshire | 9.6 (9-10.2) |
| New Jersey | 8.4 (8.2-8.6) |
| New Mexico | 4.7 (4.4-5.1) |
| New York | 8.4 (8.3-8.6) |
| North Carolina | 9 (8.8-9.2) |
| North Dakota | 10.1 (9.3-10.9) |
| Ohio | 13 (12.8-13.2) |
| Oklahoma | 11.2 (10.8-11.6) |
| Oregon | 8.7 (8.4-9) |
| Pennsylvania | 10.3 (10.1-10.5) |
| Rhode Island | 11.9 (11.2-12.6) |
| South Carolina | 7.7 (7.4-8) |
| South Dakota | 9.1 (8.4-9.8) |
| Tennessee | 10.9 (10.6-11.2) |
| Texas | 9 (8.8-9.1) |
| Utah | 2.5 (2.3-2.8) |
| Vermont | 13.1 (12.1-14) |
| Virginia | 6.4 (6.2-6.6) |
| Washington | 7.9 (7.7-8.1) |
| West Virginia | 15.4 (14.8-15.9) |
| Wisconsin | 7.6 (7.3-7.8) |
| Wyoming | 6.4 (5.6-7.1) |

Supplemental Table 7: Complications of Lung Cancer and Ischemic Heart Disease–related Age-Adjusted Mortality Rates per 100,000, Stratified by Census Region in Adults in the United States, 1999 to 2020

| **Census Region** | **Year** | **Age-Adjusted Rate (95% CI)** |
| --- | --- | --- |
| Northeast | 1999 | 11.5 (11-11.9) |
| Northeast | 2000 | 11.6 (11.2-12.1) |
| Northeast | 2001 | 11.3 (10.9-11.8) |
| Northeast | 2002 | 11.6 (11.1-12) |
| Northeast | 2003 | 11.4 (10.9-11.8) |
| Northeast | 2004 | 10.8 (10.4-11.3) |
| Northeast | 2005 | 10.8 (10.4-11.3) |
| Northeast | 2006 | 10.6 (10.1-11) |
| Northeast | 2007 | 10.2 (9.8-10.6) |
| Northeast | 2008 | 9.6 (9.2-10) |
| Northeast | 2009 | 9.4 (9-9.8) |
| Northeast | 2010 | 9.5 (9.1-9.9) |
| Northeast | 2011 | 9.1 (8.7-9.5) |
| Northeast | 2012 | 8.7 (8.3-9.1) |
| Northeast | 2013 | 8 (7.6-8.3) |
| Northeast | 2014 | 7.6 (7.2-7.9) |
| Northeast | 2015 | 6.9 (6.5-7.2) |
| Northeast | 2016 | 6.5 (6.1-6.8) |
| Northeast | 2017 | 6.2 (5.9-6.5) |
| Northeast | 2018 | 5.9 (5.6-6.2) |
| Northeast | 2019 | 5.7 (5.4-6) |
| Northeast | 2020 | 5.8 (5.5-6.1) |
| Northeast | **Total** | 8.8 (8.7-8.9) |
| Midwest | 1999 | 11.6 (11.1-12) |
| Midwest | 2000 | 10.9 (10.5-11.4) |
| Midwest | 2001 | 11.2 (10.7-11.6) |
| Midwest | 2002 | 11.1 (10.7-11.6) |
| Midwest | 2003 | 11.3 (10.8-11.7) |
| Midwest | 2004 | 11.4 (11-11.9) |
| Midwest | 2005 | 11.4 (11-11.8) |
| Midwest | 2006 | 11.3 (10.9-11.7) |
| Midwest | 2007 | 11.2 (10.8-11.6) |
| Midwest | 2008 | 10.9 (10.4-11.3) |
| Midwest | 2009 | 10.3 (9.9-10.7) |
| Midwest | 2010 | 10.1 (9.7-10.5) |
| Midwest | 2011 | 9.9 (9.5-10.3) |
| Midwest | 2012 | 9.5 (9.2-9.9) |
| Midwest | 2013 | 8.5 (8.2-8.9) |
| Midwest | 2014 | 8.6 (8.3-9) |
| Midwest | 2015 | 7.8 (7.4-8.1) |
| Midwest | 2016 | 7.6 (7.2-7.9) |
| Midwest | 2017 | 7.5 (7.2-7.8) |
| Midwest | 2018 | 7.3 (7-7.6) |
| Midwest | 2019 | 7.1 (6.8-7.4) |
| Midwest | 2020 | 7.6 (7.3-7.9) |
| Midwest | **Total** | 9.6 (9.5-9.7) |
| South | 1999 | 9.9 (9.5-10.2) |
| South | 2000 | 10 (9.7-10.3) |
| South | 2001 | 9.9 (9.6-10.3) |
| South | 2002 | 10.2 (9.8-10.5) |
| South | 2003 | 9.6 (9.3-9.9) |
| South | 2004 | 9.4 (9.1-9.8) |
| South | 2005 | 10 (9.7-10.3) |
| South | 2006 | 9.4 (9.1-9.8) |
| South | 2007 | 9.2 (8.9-9.5) |
| South | 2008 | 8.9 (8.6-9.2) |
| South | 2009 | 8.9 (8.7-9.2) |
| South | 2010 | 9.1 (8.8-9.3) |
| South | 2011 | 8.3 (8-8.6) |
| South | 2012 | 8.1 (7.8-8.4) |
| South | 2013 | 7.9 (7.6-8.1) |
| South | 2014 | 7.1 (6.9-7.4) |
| South | 2015 | 7 (6.8-7.3) |
| South | 2016 | 7.2 (6.9-7.4) |
| South | 2017 | 7 (6.7-7.2) |
| South | 2018 | 7 (6.8-7.3) |
| South | 2019 | 7 (6.8-7.2) |
| South | 2020 | 7.4 (7.2-7.7) |
| South | **Total** | 8.4 (8.3-8.5) |
| West | 1999 | 9.7 (9.3-10.1) |
| West | 2000 | 9.1 (8.7-9.5) |
| West | 2001 | 9.2 (8.8-9.6) |
| West | 2002 | 8.7 (8.3-9.2) |
| West | 2003 | 8.8 (8.4-9.3) |
| West | 2004 | 8.2 (7.8-8.5) |
| West | 2005 | 8.4 (8-8.8) |
| West | 2006 | 8.4 (8-8.7) |
| West | 2007 | 7.8 (7.4-8.2) |
| West | 2008 | 7.2 (6.9-7.6) |
| West | 2009 | 7.2 (6.9-7.6) |
| West | 2010 | 7.6 (7.3-8) |
| West | 2011 | 7 (6.6-7.3) |
| West | 2012 | 6.4 (6.1-6.7) |
| West | 2013 | 6.1 (5.8-6.4) |
| West | 2014 | 5.8 (5.6-6.1) |
| West | 2015 | 5.3 (5-5.6) |
| West | 2016 | 5.1 (4.8-5.3) |
| West | 2017 | 4.9 (4.7-5.2) |
| West | 2018 | 4.7 (4.5-4.9) |
| West | 2019 | 4.8 (4.6-5.1) |
| West | 2020 | 5 (4.8-5.3) |
| West | **Total** | 6.8 (6.7-6.9) |
| **Total** | **Total** | 8.4 (8.3-8.4) |

| **Age-Adjusted Rate (95% CI)** | | |
| --- | --- | --- |
| **Year** | **Metropolitan** | **Nonmetropolitan** |
| 1999 | 10.4 (10.2-10.6) | 11.4 (10.9-11.9) |
| 2000 | 10.2 (9.9-10.4) | 11.4 (10.9-11.9) |
| 2001 | 10.1 (9.9-10.4) | 11.3 (10.8-11.8) |
| 2002 | 10 (9.8-10.2) | 12.3 (11.8-12.8) |
| 2003 | 9.8 (9.6-10.1) | 11.7 (11.3-12.2) |
| 2004 | 9.5 (9.3-9.7) | 11.9 (11.4-12.4) |
| 2005 | 9.8 (9.6-10) | 11.7 (11.2-12.2) |
| 2006 | 9.4 (9.2-9.6) | 11.8 (11.3-12.3) |
| 2007 | 9.1 (8.9-9.3) | 11.6 (11.1-12) |
| 2008 | 8.6 (8.4-8.8) | 11.3 (10.8-11.8) |
| 2009 | 8.5 (8.3-8.7) | 11.2 (10.7-11.6) |
| 2010 | 8.7 (8.5-8.9) | 10.9 (10.4-11.3) |
| 2011 | 8.1 (8-8.3) | 10.3 (9.8-10.7) |
| 2012 | 7.8 (7.6-7.9) | 10 (9.6-10.4) |
| 2013 | 7.3 (7.1-7.4) | 9.4 (9-9.9) |
| 2014 | 6.8 (6.7-7) | 9.2 (8.8-9.6) |
| 2015 | 6.3 (6.2-6.5) | 9.2 (8.8-9.6) |
| 2016 | 6.2 (6.1-6.4) | 8.8 (8.4-9.1) |
| 2017 | 6.1 (5.9-6.2) | 8.6 (8.2-8.9) |
| 2018 | 5.9 (5.7-6) | 8.7 (8.3-9) |
| 2019 | 5.9 (5.8-6.1) | 8.2 (7.8-8.5) |
| 2020 | 6.1 (6-6.2) | 9.5 (9.1-9.8) |
| Total | 8 (8-8) | 10.3 (10.2-10.4) |

Supplemental Table 8: Complications of Lung Cancer and Ischemic Heart Disease– related Age-Adjusted Mortality Rates per 100,000, Stratified by Urban-Rural Classification in Adults in the United States, 1999 to 2020
